# The Expression of Markers for Intratubular Germ Cell Neoplasia in Normal Infantile Testes

**DOI:** 10.3389/fendo.2018.00286

**Published:** 2018-06-01

**Authors:** Kolja Kvist, Erik Clasen-Linde, Oline Langballe, Steen Holger Hansen, Dina Cortes, Jorgen Thorup

**Affiliations:** ^1^The Department of Pediatric Surgery, Copenhagen University Hospital Rigshospitalet, Copenhagen, Denmark; ^2^The Department of Pathology, Copenhagen University Hospital Rigshospitalet, Copenhagen, Denmark; ^3^Department of Forensic Medicine, University of Copenhagen, Copenhagen, Denmark; ^4^Department of Pediatrics, Section of Endocrinology, Copenhagen University Hospital Hvidovre, Hvidovre, Denmark; ^5^Faculty of Health and Medical Sciences, University of Copenhagen, Copenhagen, Denmark

**Keywords:** testis maturation, testis, immunohistochemistry, germ cells, testicular neoplasms

## Abstract

**Background:**

Positive immunohistochemical expression of testicular cancer markers is often reported beyond 12 months of age in cryptorchid testes, which is assumed to indicate delayed maturation of the fetal germ cells, or neoplastic changes. These findings allowed for questions as to the extent of positive reaction in normal testes. The aim of the study was to clarify the expression of these markers in a normal material up to 2 years.

**Methods:**

Testicular material from 69 boys aged 1–690 days, who died of causes with no association of testicular pathology. Histology sections were incubated with primary antibodies including anti-placental-like alkaline phosphatase (PLAP), anti-C-Kit, anti-D2–40, and anti-Oct3/4. The mean germ cell number per tubular transverse section (G/T) was calculated based on the G/T of both testes of every boy.

**Results:**

The mean G/T declined through the 690 days. PLAP appeared stably expressed throughout the ages studied. The likelihood of a positive reaction for C-Kit waned with increasing age within the study period. Positive staining for D2–40 and Oct3/4 was demonstrated up to 6 and 9 months respectively.

**Conclusion:**

Up to 1 or 2 years of age, normal infantile testes contain germ cells positive for the immunohistochemical markers commonly utilized to aid in the detection of testicular cancer. This finding supports the concept of germ cells undergoing a continuous maturational process in a heterogeneous fashion, and that this process is not complete by 2 years of age.

## Introduction

Intratubular germ cell neoplasia (ITGCN) is a recognized precursor of invasive testicular cancer (1). In adults it has a well-described histological appearance with atypical germ cells lined adjacent to a thickened basement membrane inside the seminiferous tubuli. The cells are large, have large nuclei with a hyperchromatic, coarse chromatin pattern, large prominent nucleoli, and abundant pale cytoplasm. In prepubertal boys the cells are morphologically similar, but located both centrally and peripherally, and the basal membrane is not thickened (2).

Commonly, the detection of ITGCN in adults is aided by the application of immunohistochemistry with antibodies toward the receptors for placental-like alkaline phosphatase (PLAP), C-Kit, D2–40, and Oct3/4. The sensitivity and specificity of detecting ITGCN in adults, using these markers, is high (3).

The observation that fetal germ cells express these same markers funneled the hypothesis of ITGCN originating from fetal germ cells failing to mature properly (4).

Cryptorchidism—when one, or both, testis has failed to descend into the scrotum—is a congenital condition, where there is evidence of disruption in spermatogenesis (2). It is among the more common congenital defects, for which 2–3% of boys in the western world are operated. Boys with cryptorchidism have an associated relative risk of developing testis cancer ranging from 3.7 to 7.5 times higher than in normal boys (5). Cryptorchidism also confers a risk of infertility ranging from 10% in unilateral cases to 70% in those bilaterally afflicted (2, 6, 7).

We recently published our findings of positive immunostaining of spermatogonia in biopsies from 404 cryptorchid boys with PLAP, C-Kit, D2–40, and Oct3/4, aged up to 15 years without histological features of concomitant cancer or ITGCN, except for two boys. One boy 13 months old was diagnosed with Prader–Willi syndrome and had been treated with growth hormone; the other was 44 months old with 45X/46XY, penoscrotal hypospadia, persistent vaginal, and uterine remnants, and both testes located intra-abdominally (8, 9). This is consistent with ITGCN in a previously published cohort of cryptorchid boys of around 0.5% (7/1403) (10). Conversely, in a cohort study from our region, the incidence of testicular cancer in adults treated for cryptorchidism in childhood was 1.2% (6 out of 506 subjects), and in general around 5% of testicular cancers are statistically referred to cryptorchidism (5, 11).

Thus in relation to cryptorchidism, it does not immediately follow, that a subsequent development of ITGCN or testicular cancer resides within dormant, dysmature spermatogonia. Moreover, screening by way of immunohistochemistry, can not stand alone in cryptorchid boys.

These findings naturally allowed for questions as to the extent of positive reaction in normal testes, and after reviewing the literature, we found very few studies regarding human tissue beyond the neonatal period. Most studies have been done on tissue from aborted fetuses and/or stillborn children and from adults (4, 12–20). From these it would appear that in normal testes the germ cells become negative for these immunohistochemical markers after the first year of age. Therefore, our previous findings of a positive reaction to the same markers in undescended testes beyond this age support the prevailing concept of delayed maturation of spermatogonia within cryptorchid testes (8).

Thus, this study aims to substantiate the hypothesis: that the pool of spermatogonia is heterogeneous and continues to transform and differentiate after birth; that this is demonstrable through the varying expression of different immunohistochemical markers in neonatal and infantile testes; and that we hereby can strengthen the concept of delayed maturation in the cryptorchid testis.

This process would also follow an evaluation of the timing of the disappearance of a positive reaction to the aforementioned panel of antibodies in normal infantile testes.

## Materials and Methods

From the Department of Forensic Medicine was acquired testicular material from the testes of 69 boys, aged 1–690 days (mean and median 360 days), who died of causes with no known association of testicular pathology.

The tissue had been fixed in 4% formalin. The exact ischemia time was unknown, but estimated to be between 1 and 5 days.

Due to scarcity of tissue, tissue microarrays (TMA) of the paraffin-embedded biopsies were performed, with two cores from each biopsy. The resulting block was cut in 2 µm sections and mounted on coated slides (Dako Flex IHC microscope slides) for immunohistochemical analysis, which was performed on Ventana Benchmark Ultra Stainer.

One hematoxylin-eosin (H + E) slide was produced.

For immunohistochemistry sections were dewaxed with EZ-prep from Ventana. Antigen-retrieval slides were pretreated with Ventana CDI (cell conditioning pH 8.5) for 64 min and incubated with antibodies for 32 min at 36°C following the manufacturer’s instructions. The reaction was visualized using Ventana Ultra View DAB-kit. Counterstaining was with Ventana hematoxylin for 8 min.

Histology sections were incubated with primary antibodies, including anti-PLAP (1:200, PL8-F6, Biogenex), anti-C-Kit receptor (1:50, C-Kit, Dako, Glostrup, Denmark), D2–40 (1:25, M3619, Dako, Glostrup, Denmark), and anti-Oct3/4 (RTU, MRG, Cellmarque, CA, USA).

The number of germ cells per tubular transverse section was measured from at least 30 tubular transverse sections (G/T) from testes and the mean G/T was calculated for each boy.

For identification of germ cells with positive reaction to antibody, all seminiferous tubuli were examined by two observers. In cases where positive signals from germ cells were seen in some seminiferous tubules only, this focal expression was classified as a positive result.

Both the counting and identification was done in blinded fashion, and only when the results were established, was the identity of the boy revealed.

We evaluated the timing of the disappearance of a positive reaction to the aforementioned panel of antibodies in these normal infantile testes.

Statistical analysis was performed using SAS University Edition.

The study was conducted according to the Helsinki II declaration and approved by the ethics committee of Copenhagen (Protocol #H-3-2010-074).

## Results

From the records, we extracted age, weight, and cause of death. The birth weight was only available in less than one-third of cases, and gestational age not at all. The causes of death were infection, congenital heart disease, suffocation, homicide, trauma, and unknown.

In the H + E stained sections it was possible to discriminate between Sertoli cells and germ cells, but not to positively discern fetal spermatogonia from adult dark (Ad) and adult pale (Ap) spermatogonia, or between the latter two, due to edema and clustering of the cells centrally within the tubuli, rendering it also impossible to discern whether the germ cells were centrally or peripherally located.

**Figure 1:**
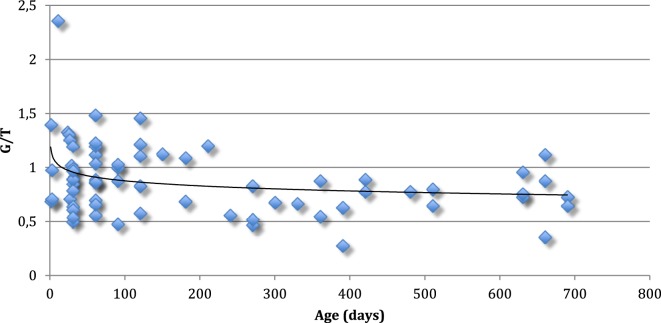
Mean germ cell number per tubular cross-section (G/T) of the testes from 69 normal boys up to 2 years of age. Every boy is represented with one value. The line represents median G/T for every age.

The mean G/T of the testes from every boy is shown in Figure 1.

The results of the immunostaining are summarized in Table 1. Regarding the positive markers there was concordance between the two testes of the same boy.

**Table 1:** Number of boys with a positive reaction to each the immunohistochemical markers grouped by age in months.

Age	*n*(boys)	Placental-like alkaline phosphatase	Oct3/4	C-Kit	D2–40
0–1 month	10	10/10 (100%)	10/10 (100%)	10/10 (100%)	8/10 (80%)
1–3 months	22	22/22 (100%)	15/22 (68%)	18/22 (82%)	12/22 (55%)
3–6 months	10	10/10 (100%)	6/10 (60%)	8/10 (80%)	4/10 (40%)
6–9 months	4	4/4 (100%)	1/4 (25%)	3/4 (75%)	0
9–12 months	6	6/6 (100%)	0	4/6 (67%)	0
12–15 months	6	6/6 (100%)	0	3/6 (50%)	0
15–18 months	3	3/3 (100%)	0	2/3 (67%)	0
18–21 months	0	NA	NA	NA	NA
21–24 months	8	8/8 (100%)	0	3/8 (38%)	0

*NA, no boys within this age group*.

Placental-like alkaline phosphatase appeared stably expressed throughout the ages studied. The likelihood of a positive reaction for C-Kit waned with increasing age, but remained positive until 2 years of age. Statistical analysis with regression analysis on the percentage of positive staining confirmed this. Positive staining for D2–40 and Oct3/4 was demonstrated up to 6 and 9 months, respectively. In the groups 1–3, 3–6, and 6–9 months all D2–40 positive testes were also positive for the other three markers and those who were D2–40 negative but Oct3/4 positive were also C-Kit and PLAP positive (Table 1). For all the other groups the results regarding positive staining were unambiguous (Table 1).

There was a sense, between the observers, that the intensity of staining was also inversely related to age. However, as the biopsies were obtained from autopsies and processed by way of TMA, the general staining pattern was quite heterogeneous and no attempt was made to quantify the intensity.

**Figure 2:**
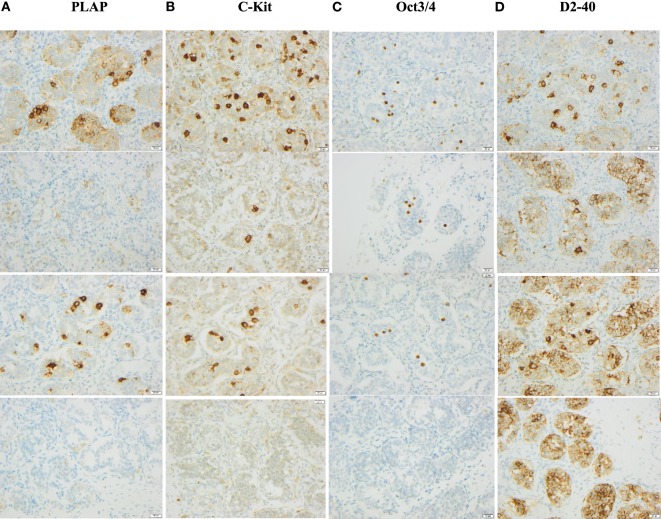
Examples of staining for immunohistochemical markers in testicular biopsies of boys 10, 30, 60, and 660 days old, respectively. Column A shows PLAP staining in age order starting with the youngest age in upper row. Column B shows C-Kit staining in the same age order. Column C shows Oct3/4 staining in the same age order. Column D shows D2–40 staining in the same age order.

Figure 2 shows positive staining for the immunohistochemical markers in testicular biopsies.

## Discussion

Our results are somewhat surprising. To our knowledge this is the first time a positive staining by PLAP and C-Kit has been reported beyond 12 months of age in normal testes.

We had expected to confirm, what others had found before, namely that there would be no positive reaction beyond 12 months of age (4, 12, 21). However, when the maturation of the primordial germ cells (PGC’s) through sequential steps into adult spermatogonia is viewed as a continuum, our results are not in conflict. They merely suggest that the maturation is neither uniform nor complete at 2 years of age.

Previous studies with normal fetuses and neonates have evaluated the expression of several different immunohistochemical markers of differentiation and stem cell-ness. Some of these are also used to detect ITGCN (13, 20), and hence have supported the concept of ITGCN originating in fetal life.

Jørgensen and colleagues in 1993 and 1995 reported that in normal infantile testis the expression of PLAP was not detected beyond 12 months of age (4, 12). This was later confirmed and also applicable for C-Kit and Oct3/4 by Vigueras-Villasenor et al. in 2015 (21). They reported that C-Kit and Oct3/4 positive germ cells were not seen after 4 months of age and PLAP positive germ cells were seen in newborns until 1 year of age (21). Others have found similarly diminished to absent expression of the other markers of the panel in normal boys (13, 20). Rajpert-De Meyts et al. (13) found that the oldest specimen in their series with a few nuclei weakly positive for OCT3/4 was from a 4 months old infant. Thereafter, all prepubertal, peripubertal, and adult testicular samples in their series of normal males were consistently negative. However most authors have only included gonads from fetuses and neonatal boys (14–17, 20).

The fact, that we find much stronger expression of particularly PLAP and C-Kit in the present normal testes, as previously shown in cryptorchid testes (8), could be ascribed to enhanced staining due to technical refinement both with regards to staining procedure and antibody production.

Taken together, the studies of the literature suggest that once the PGC’s have arrived from the allantois to the gonadal ridge by week 6 of gestation and become enclosed in the newly formed seminiferous tubuli they continue to differentiate and proliferate, but not in unison (22). Rather they make up a heterogeneous pool of gonocytes at different differential stages, gradually maturing into fetal spermatogonia. This is evidenced by their spatial expression of the varying immunohistochemical markers (4, 12–18, 20), their morphology (16, 17) and localization (14, 16, 17) within the tubuli as showed in Table 2.

**Table 2:** Previously reported immunohistochemical staining of primordial germ cells (PGC’s) and germ cells in fetuses and neonates.

	PGC	1st trimester	2nd trimester	3rd trimester	Neonatal
Placental-like alkaline phosphatase	+	+++	++	+	+/−
OCT3/4	+	+++	++	+	+/−
C-Kit	+	+++	++	+	+/−
D2–40	?	+	++	++	+
AP-2γ	+	++	++	+	+/−
MAGE-A4	–	+	++	+++	++
VASA	+/−	+	++	+++	++
TSPY	?	+	++	+++	+++
Ki-67	?	?	+++	++	++

*+, positive reaction; ++, intermediate positive reaction; +++, strong positive reaction; −, negative reaction; ?, unclassified. Data compiled from Jørgensen et al. (4, 12), Hoei-Hansen et al. (18), Robinson et al. (15), Gaskell et al. (16), Rajpert-de Meyts et al. (13), Pauls et al. (17), Honecker et al. (20), and Franke et al. (14)*.

The D2–40 antibody recognizes the M2A antigen, a marker for adult ITGCN and seminoma. Downregulation of D2–40 expression coincides with the movement of gonocytes toward the basement membrane as they lose embryonic stem-cell-like phenotype and differentiate into spermatogonia according to Sonne et al. (23). Those D2–40 positive germ cells were the first to disappear in our material and are, therefore, likely gonocytes. Oct3/4 positive germ cells in this age group represent other early stages of fetal germ cell maturation (possibly gonocytes at other differential stages and pre-spermatogonia) which normally proceeds into the neonatal age and early infancy (8, 20).

The process continues, and gradually more Ad and Ap spermatogonia appear according to the literature (2, 6). Of these Ad spermatogonia has been regarded as the “reserve” stem cell and Ap spermatogonia as the “active” stem cell, dividing once every epithelial cycle and eventually giving rise to type B spermatogonia (24). The PLAP and C-Kit positive staining in our material may likely represent persisting germinative stem cell properties.

Particularly the period of mini-puberty—a term coined for the phase of transient rise in gonadotropins and androgens that sets in between 2 and 4 months of life—seems to be crucial in driving the maturation of the germ cells (7, 25). In some cryptorchid boys, this hormonal activation may be a stunted, as evidenced by a transient hypothalamus–pituitary–gonadal hypo-function seen at surgery for the undescended testis (26). Because of the forensic medicine character of the material we had no possibility to determine serum levels of reproductive hormones (gonadotropins, androgens, and inhibin-B). It would have been interesting to relate the results of such blood samples to the immunohistochemical staining pattern of germ cells, especially during the period of mini-puberty.

In cryptorchid testes a diminished number of spermatogonia—both Ad and Ap—are particularly described, and we have previously reported positive reaction to D2–40, and Oct3/4 upto 19 months of age (6–8).

These findings—diminished number of spermatogonia, stunted mini-puberty, and positive reaction to the above panel of markers—have supported the prevailing concept of delayed maturation of germ cells in cryptorchid testes.

**Figure 3:**
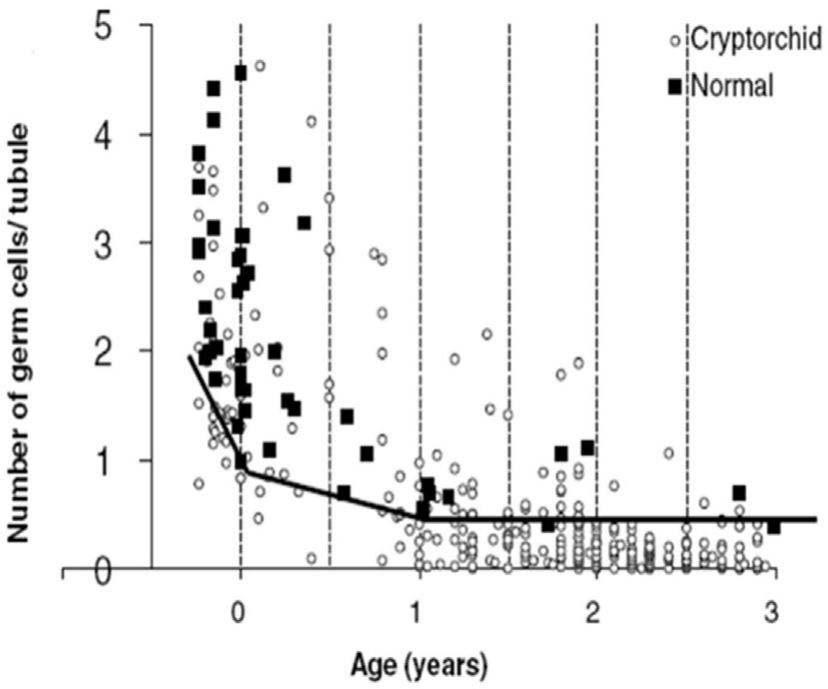
The number of germ cells (spermatogonia and gonocytes if any) per tubular cross-section (G/T) in human cryptorchid fetuses, and in boys who underwent surgery for cryptorchidism <3 years of age. Furthermore, G/T in normal human fetuses and normal boys who died <3 years of age. The solid line indicates the lowest normal G/T. Data with permission from Cortes (31) and Cortes et al. 1995 (32).

Our findings of the aforementioned positive immunohistochemical reactions in normal testis upto 2 years of age, suggest that there is both a maturational delay in cryptorchid testis and an accelerated loss of spermatogonia (27–29). Indeed, D2–40 and Oct3/4 positive germ cells persist in cryptorchid testes into the second year of life and PLAP and C-Kit positive germ cells persist with almost the same frequency as in our material of normal testes (30) (Table 3). The decline in mean G/T with age in this study is in accordance with previous findings reported (2, 31) (Figures 1 and 3). However, when compared to our previously published normal material a significant loss of germ cells in cryptorchid testes is seen already 1 year of age (31, 32) (Figure 3).

**Table 3:** Percentage of testes with a positive reaction to each of the immunohistochemical markers grouped by age in years.

Age	*n*(boys)	*n*(testes)	Placental-like alkaline phosphatase (PLAP) pos (%)	Oct3/4 pos (%)	C-Kit pos (%)	D2–40 pos (%)
**Testes from normal boys. Present forensic medicine material**						
0–<1/2 years	42	84	100	73	86	57
1/2–<1 years	10	20	100	10	70	0
1–<2 years	17	34	100	0	47	0

**Testes from boys with cryptorchidism. Materials from Thorup et al. (30)**						
0–<1/2 years	33	43	98	91	93	54
1/2–<1 years	189	220	95	59	70	23
1–<2 years	249	324	89	12	33	4

This loss could be attributed to increased apoptosis, which in turn could be ascribed to decreased hormonal values and the increased temperature, the retained testis is subjected to Ref. (27–29). Several studies have described the deleterious effect of increased temperature on the testes and the displacement of a testis to the abdomen in experimental settings often result in a Sertoli-cell-only pattern. In intra-abdominally located cryptorchid testis biopsied at the time of surgery, less than 10% contain germ cells after the age of 3 years (33, 34).

We do not know if any of the boys in our material would eventually have developed testicular cancer. However, our findings do question the hypothesis that such cancers in general develop from dormant fetal germ cells, as it raises the age at which PLAP and C-Kit positive germ cells with stem cell properties should no longer be present. In this study, PLAP and C-Kit positive germ cells are only reported up to 2 years of age. But this information may be added to the findings that PLAP-positive cells were seen in 57–82% and c-Kit-positive in 5–21% of cryptorchid testes between 4 and 13 years, who unlikely would develop cancer (30). Also, in contrast, among 11 men with testicular cancer 24–37 years old previously operated for non-syndromic cryptorchidism in childhood only one had PLAP positive germ cells in the prepubertal biopsy. In all the others all staining were negative except for one case that had Oct3/4 and D2–40 positive cells in the prepubertal biopsy and developed a teratocarcinoma 27 years old. But a teratoma anlage is known to be congenital precursor to cancer (10).

One weakness of this study is the poor histological preservation of the tissue, rendering finer observations impossible, as mentioned above. Another weakness, when making comparison to the other studies, is that we used TMA, making quantification of the immunohistochemical reactions unreliable; being heterogeneous in a section of a biopsy of a biopsy. Finally, but not least, we are unable to say when the maturation of germ cells is complete. Or at which age a positive reaction to the above panel of markers is pathological, as we only studied testes in boys up to 2 years of age. Alas, this age was chosen, as we had expected to confirm their disappearance at 12 months.

## Conclusion

We showed that normal infantile testes contain germ cells positive for PLAP a C-Kit commonly utilized to aid in the detection of ITGCN upto 2 years of age. Positive staining for D2–40 and Oct3/4 was demonstrated up to 6 and 9 months, respectively. Therefore, a positive reaction to these antibodies is not diagnostic of neoplastic changes in this age group, but reflects that the normal germ cell maturational process is not completed by the age of 2 years.

Compared to our previous findings of a positive reaction to the same immunohistochemical markers in undescended testes beyond this age, our new findings confirm the hypothesis of delayed maturation of spermatogonia within cryptorchid testes.

## Ethics Statement

The study was conducted according to the Helsinki II declaration and approved by the ethics committee of Copenhagen (Protocol #H-3-2010-074). As the individuals from where the biopsies were taken have passed away and were anonymous and taken for forensic medicine legal causes the ethical committee accepted that no consent was needed.

## Author Contributions

KK did germ cell evaluation and wrote first draft of manuscript. EC-L stained biopsies, evaluated germ cells, supervised cell counting and manuscript. OL wrote protocol of study, applied for ethical consent. SH took all biopsies of the material, supervised manuscript. DC planned the study and revised manuscript. JT planned the study and revised manuscript All authors accepted the final manuscript. None of the authors have any disclosures.

## Conflict of Interest Statement

The authors declare that the research was conducted in the absence of any commercial or financial relationships that could be construed as a potential conflict of interest. The handling Editor declared a past co-authorship with the author JT.
